# Photoaccelerated Water Dissociation Across One-Atom-Thick Electrodes

**DOI:** 10.1021/acs.nanolett.2c03701

**Published:** 2022-11-30

**Authors:** Junhao Cai, Eoin Griffin, Victor Guarochico-Moreira, Donnchadh Barry, Benhao Xin, Shiqi Huang, Andre K. Geim, Francois. M. Peeters, Marcelo Lozada-Hidalgo

**Affiliations:** † National Graphene Institute, The University of Manchester, Manchester M13 9PL, U.K.; ‡ Department of Physics and Astronomy, The University of Manchester, Manchester M13 9PL, U.K.; § College of Advanced Interdisciplinary Studies, National University of Defense Technology, Changsha, Hunan 410073, China; ∥ Escuela Superior Politécnica del Litoral, ESPOL, Facultad de Ciencias Naturales y Matemáticas, P.O. Box 09-01-5863, Guayaquil, Ecuador; ⊥ Departement Fysica, Universiteit Antwerpen, Groenenborgerlaan 171, B-2020 Antwerp, Belgium

**Keywords:** 2D materials, water dissociation, graphene, photoproton effect

## Abstract

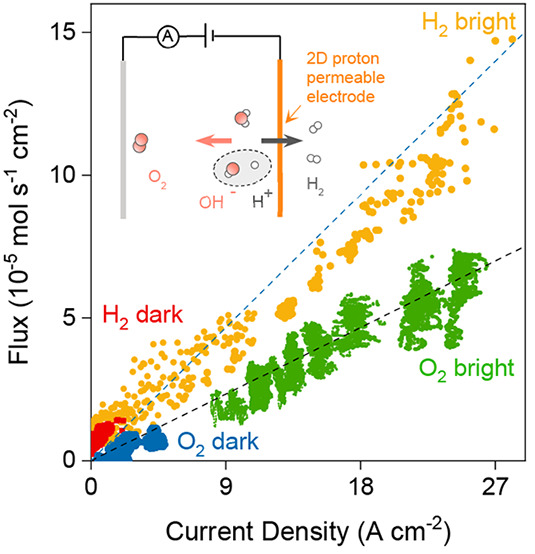

Recent experiments demonstrated that interfacial water dissociation (H_2_O ⇆ H^+^ + OH^–^) could be accelerated exponentially by an electric field applied to graphene electrodes, a phenomenon related to the Wien effect. Here we report an order-of-magnitude acceleration of the interfacial water dissociation reaction under visible-light illumination. This process is accompanied by spatial separation of protons and hydroxide ions across one-atom-thick graphene and enhanced by strong interfacial electric fields. The found photoeffect is attributed to the combination of graphene’s perfect selectivity with respect to protons, which prevents proton–hydroxide recombination, and to proton transport acceleration by the Wien effect, which occurs in synchrony with the water dissociation reaction. Our findings provide fundamental insights into ion dynamics near atomically thin proton-selective interfaces and suggest that strong interfacial fields can enhance and tune very fast ionic processes, which is of relevance for applications in photocatalysis and designing reconfigurable materials.

A unique combination of properties in graphene allows using it as a proton-permeable electrode. One-atom-thick graphene exhibits high in-plane electric conductivity^1^ and relatively easy proton transport through its basal plane.^2−4^ It is also impermeable to all atoms^5−7^ and all other ions^8,9^ and has exceptional mechanical strength.^10^ A recent work reported interfacial water dissociation^11^ (H_2_O ⇆ H^+^ + OH^–^) through graphene electrodes.^12^ These electrodes allow measuring the intrinsic proton currents arising exclusively from the dissociation reaction while experimentally monitoring the interfacial electric field, *E*. The proton currents were found to be exponentially accelerated with increasing *E* (that reached above 10^8^ V m^–1^), a phenomenon known as the Wien effect. In particular, graphene’s perfect selectivity with respect to protons and its atomic thickness were crucial to observe the Wien effect. These properties enable the intense interfacial *E* to separate protons from OH^–^ ions across the atomically thin barrier that prevents their recombination, thus yielding notable proton currents. The time scale of the involved separation process should be extremely fast—as a first approximation, comparable to the time scale of proton transport and proton–OH^–^ recombination in water, which is in the subpicosecond range.^13^ On the other hand, previous experiments showed that proton transport through graphene electrodes is strongly enhanced under illumination via a hot-electron-mediated mechanism, the so-called photoproton effect.^14^ Hot electrons in graphene have a lifetime of ∼1 ps.^15,16^ If the proton-hydroxide ion separation across graphene is comparatively fast, then in principle the photoproton effect should also accelerate the transport of protons generated by interfacial water dissociation. In this work, we report such an acceleration.

Proton-permeable graphene electrode devices were fabricated using monocrystalline graphene obtained by mechanical exfoliation, as reported previously.^2,14^ In brief, the crystals were suspended over holes (10 μm in diameter) etched in silicon-nitride substrates.^2^ The resulting graphene film was electrically connected to allow for its use as an electrode (Figure 1 and Figure S1). One side of the suspended graphene was decorated with Pt nanoparticles deposited via electron beam evaporation, which served to increase graphene’s proton conductivity.^2^ The opposite side of the suspended graphene electrode faced a 1 M KCl electrolyte with alkaline pH solution (typically, pH 11). The high KCl concentration ensures that the electrolyte resistivity is negligible, whereas the alkaline pH ensured that water is the only source of protons in the system. Hence, in this setup all proton currents arise from the water dissociation reaction, as demonstrated previously.^12^ The inner side of the devices was also coated with an anion-exchange polymer (FAA FumaTech) which is an excellent OH^–^ ion conductor.^17^ The polymer coating was not essential for the described experiments; however, it provided additional mechanical support for the membrane, improving the devices’ reliability (“Device fabrication” in Supporting Information). For electrical measurements, the devices were connected in an electrical circuit as shown in Figure 1a, using a Pt counter electrode and a silver/silver-chloride reference electrode. All potentials below are referred against the latter electrode, unless stated otherwise. Measurements were carried out inside a chamber with Ar environment, and the electrolyte was saturated with Ar to avoid a parasitic oxygen reduction reaction (“Electrical measurements” in Supporting Information).

**Figure 1:**
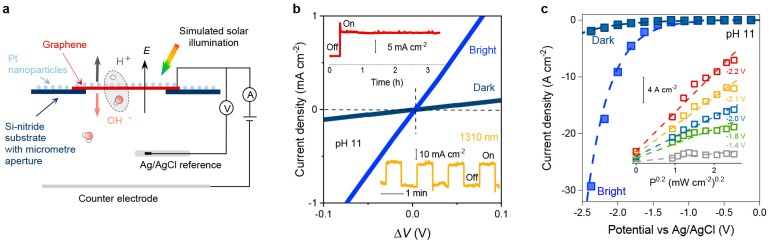
Photoeffect in water dissociation at graphene electrodes. (a) Schematic of graphene devices and the measurement setup. Water molecules dissociate under the strong interfacial *E*. Protons transfer through graphene and adsorb on its Pt-decorated external surface, whereas OH^–^ ions drift into the bulk electrolyte. Red and white balls represent oxygen and hydrogen atoms. (b) Typical *I*-Δ*V* characteristics for small Δ*V* = *V* – φ around the potential of zero current, φ (Figure S2). Response under the dark conditions (dark blue) and solar-simulated illumination of 100 mW cm^–2^ (bright blue). Dashed lines: guides to the eye. The top inset shows that the photoresponse was stable for hours of continuous illumination (Δ*V* = 0.2 V). Bottom inset: current density vs time for illumination using on–off pulses (1310 nm light source with 17 mW cm^–2^ intensity; Δ*V* = 0.4 V). (c) Examples of *I*–*V* characteristics away from the linear regime for our devices in dark (dark blue) and bright conditions (bright blue). Dashed lines: guides to the eye. Inset: *I*(*P*) ∝ *P*^0.2^ current vs illumination power relation found for devices. Dashed lines: guides to the eye.

The rationale to measure interfacial water dissociation (H_2_O ⇆ H^+^ + OH^–^) with these devices was demonstrated in ref (12). In brief, the dissociation reaction generates protons that transport through graphene. The protons are then adsorbed on Pt nanoparticles by combining with electrons (H^+^ + e^–^ → H*@Pt) that flow into graphene through the electrical circuit. These adsorbed protons eventually escape as hydrogen molecules (2H* → H_2_; Pt catalyzes this reaction) through the discontinuous Pt film.^2^ Note that the μm-sized electrodes ensure that proton transfer through graphene dominates the resistivity in the circuit, with negligible contributions from the bulk electrolyte and counter electrode^18,19^ (“Electrical measurements” in Supporting Information). On the other hand, the photoeffect in proton transport through graphene electrodes was demonstrated in ref (14). In that work, conceptually similar devices were measured using an acidic polymer electrolyte, which unlike alkaline electrolytes contains free bulk protons. Those experiments found that illumination increased the proton transport rate through the graphene electrode via a hot-electron-mediated mechanism. In brief, the discontinuous Pt nanoparticle film in the devices yields a spatially inhomogeneous charge doping on graphene that effectively results in a multitude of in-plane p–n junctions.^14^ Illuminating such junctions in graphene is known to produce an in-plane hot-electron-mediated photovoltage via the so-called photothermoelectric effect.^14,16^ In ref (14) it was shown that in the presence of an out-of-plane source of protons (the acidic polymer electrolyte) this effect strongly accelerates proton transport through graphene’s basal plane. In the present work we exploit this effect to accelerate the transfer through graphene of protons generated by the interfacial water dissociation reaction.

The current density vs voltage (*I*–*V*) response of the devices with alkaline pH electrolyte was measured both in dark conditions and under solar-simulated illumination of 100 mW cm^–2^ intensity (Oriel Sol3A light source). We found that the potential at zero current, φ, was negative, in agreement with the previous work^12^ (Figure S2). For small applied biases *V* around this potential, the *I*–*V* response was linear, which allowed extraction of the devices’ proton conductivity, *G* = *I*/Δ*V*, where Δ*V* = *V* – φ. Figure 1 shows a typical *I*–*V* response of the devices measured at pH 11 under illumination. Surprisingly, *G* increased by an order of magnitude with respect to the dark case. The inset of Figure 1 shows that this photoresponse was stable and displayed no signs of deterioration after several hours of continuous illumination. To explore these observations further, we measured the devices using solutions with different alkaline pH (Figure S3). The absolute value of *G* in dark conditions changed with pH, in agreement with the previous report.^12^ In all cases, we have observed a strong increase in *G* under illumination.

The photoeffect was also observed at high biases, away from the linear regime. In those measurements, we fixed the potential of the graphene electrode vs the reference, measured *I* as a function of time, and then illuminated the devices in 1 min long on–off pulses. Figure 1b (top inset) shows that in this high-*V* regime the devices also displayed a strong enhancement of *I* under illumination. To characterize the effect, we measured the dependence of the photoresponse as a function of the illumination power density *P* ranging from 0.7 to 100 mW cm^–2^. Figure 1c shows that the found *I*(*P*) dependence could be described by the empirical relation *I* ∝ *P*^0.2^, which is consistent with the dependence found for the photoproton effect reported in ref (14).

To rule out any possible artifact, we performed additional measurements. Neither the polymer support nor Pt nanoparticles could yield the photoresponse reported here (Figure S6). Besides graphene, this leaves only the silicon/silicon-nitride substrate as an alternative photoabsorber. It is unlikely that the robust photoeffect we observed is due to silicon, since it is insulated from the electrical contacts by a thick 500 nm nitride layer (Figure S1). Moreover, we previously demonstrated that the photoeffect in these devices can be entirely suppressed if metals like Au or Ag were used instead of Pt nanoparticles,^14^ which would be inconsistent with silicon as the photoabsorber. Nevertheless, we characterized the photoeffect using a 1310 nm light source. Such a long wavelength cannot be absorbed by the ∼1.2 eV bandgap in silicon but is readily absorbed by graphene.^20^ We also studied these devices using an acidic pH polymer (Nafion) to study the role of this long wavelength on proton transport. Figure 1b (bottom inset) shows that the devices displayed the same strong photoresponse even with this long-wavelength light. Devices measured under alkaline pH conditions displayed the same enhancement. These observations prove that the photoeffect is a feature of graphene and its proton transport.

Our results can be understood as follows. The intense electric field at the graphene–water interface, which is of the order^12^ of 10^8^ V m^–1^, dissociates water molecules into protons and hydroxide ions (“Wien effect” in Supporting Information). The same field drives protons through graphene and hydroxide ions into the electrolyte bulk, separating the generated ion pairs across the proton-selective interface that prevents their recombination. The transported proton then adsorbs on the Pt nanoparticles on the opposite side of graphene by acquiring an electron. The role of illumination can be understood using the following two observations. First, while the absolute value of *I* depends on pH, the photoeffect always enhances *I* by a factor of ∼10 with respect to the dark case. This is the same enhancement reported in ref (14) for devices in acidic pH in which protons are free in the bulk electrolyte. Second, the illumination power dependence, *I*(*P*), reported here is the same as in ref (14). These observations are consistent with photoacceleration of proton transport events via the previously reported photoproton effect. This is possible for this reaction in graphene electrodes because the intense interfacial electric field acts over atomic-scale distances and thus achieves fast separation of the generated protons across graphene—within a time scale comparable to or shorter than the ps lifetime of hot electrons in graphene (“Time scales” in Supporting Information).

Water dissociation (H_2_O ⇆ H^+^ + OH^–^) eventually leads to full electrolysis (H_2_O → H_2_ + 1/2O_2_), producing hydrogen and oxygen gas. The gas evolution rates are much slower than the dissociation step^21^ (“Time scales” in Supporting Information). However, in our devices these reactions take place in the large Pt nanoparticle film (H_2_ evolution) and the Pt counter-electrode (O_2_ evolution). These catalytically active areas are several orders of magnitude larger in size than the graphene electrode and hence effectively behave as drain reservoirs for the ions. Because of this, gas evolution reactions are not a limiting factor in our devices,^14^ and therefore, we also expect to observe an acceleration of these reactions under illumination. To confirm this, we measured rates of H_2_ and O_2_ production directly, both in dark conditions and under illumination. For hydrogen measurements, the graphene electrode faced a vacuum chamber connected to a mass spectrometer, whereas an oxygen-concentration sensor (Clark microelectrode) placed inside the electrolyte solution monitored oxygen production (Figures S4 and S5). For zero or positive voltages applied to graphene, no H_2_ could be detected by the spectrometer, in agreement with the previous work.^6,14^ For the negative polarity, both H_2_ flux and electric current were detected simultaneously. Figure 2 shows that for every two electrons that flowed through the electrical circuit one H_2_ molecule was detected by the spectrometer. This charge-to-mass conservation is described by Faraday’s law of electrolysis: Φ_H2_ = *I*/2*F*, with Φ_H2_ the hydrogen flux and *F* the Faraday constant. For O_2_ gas, the area-normalized derivative of the oxygen concentration versus time, d(O_2_)/d*t* = Φ_O_, was also described by Faraday’s law, Φ_O_ = *I*/4*F* (Figure 2). Illuminating the devices resulted in an instantaneous increase in both electrical current and gas flux (Figures S4 and S5) that was also consistent with Faraday’s law of electrolysis. The found relations show that H_2_ and O_2_ molecules were generated in a 2:1 ratio with 100% Faradaic efficiency, which again shows that the measured currents in our devices are due to water dissociation.

**Figure 2:**
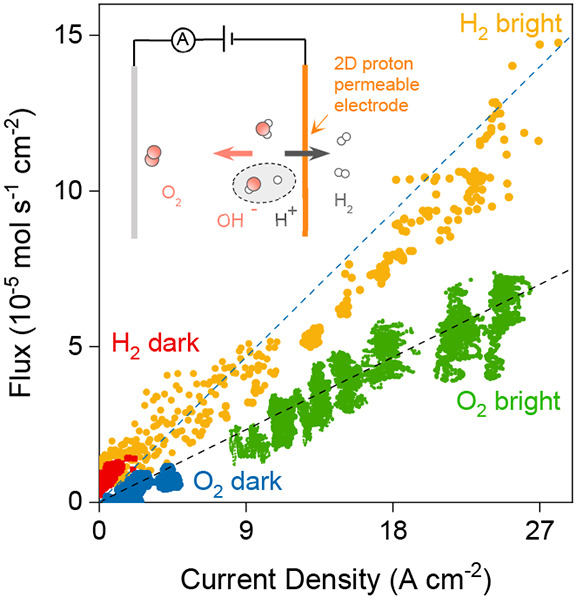
Faradaic efficiency measurements. Hydrogen and oxygen fluxes as a function of *I* under dark and bright conditions; color coded. The dotted lines correspond to Φ_H2_ = *I*/2*F* and Φ_O_ = *I*/4*F*. Inset: schematic of gas production measurements.

Our work reports a strong photoeffect in the interfacial water dissociation reaction using one-atom-thick graphene electrodes. The observation is attributed to acceleration of proton transport which happens in synchrony with the dissociation reaction. The findings are consistent with both the fast rate of proton transport and proton–OH^–^ recombination in water (subpicosecond scale) and the lifetime of hot electrons in graphene (picosecond time scale). We have also shown that strong electric fields acting across atomically thin and highly selective interfaces can enable ultrafast ion-charge separation. The fundamental insights gained here could be of interest for development of photocatalysts, for which charge separation is a central consideration. Another emerging possibility is the use of protons to reversibly modify the electronic properties of materials. This has been explored in low power memory storage,^22^ plasmonic materials,^23^ and neuromorphic hardware.^24^ The protonation dynamics in these applications is important as it can control the response time in write/read or potentiation/depotentiation cycles.^22−24^ In addition, our work suggests that atomically thin interfaces can control ion-charge separation dynamics at time scales comparable to those in optoelectronics, which could open new opportunities in these applications.

## Supporting Information Available

Detailed description of device fabrication and experimental setups; data of photoeffect measured using different electrolyte pH; real-time data of H_2_ and O_2_ gas evolution; and supplementary discussions on the Wien effect and on the time scales of the water dissociation process (PDF)
