# Supplementary material for: Photoaccelerated Water Dissociation Across One-Atom-Thick Electrodes
Source: Nano Lett. 2022 Nov 30;22(23):9566–70. doi: 10.1021/acs.nanolett.2c03701 (PMC9756329; doi:10.1021/acs.nanolett.2c03701)
Supplement: Supplementary file 1 — nl2c03701_si_001.pdf [file nl2c03701_si_001.pdf]

Supporting Information for

Photo-accelerated water dissociation across one-atom-thick electrodes

Junhao Cai[1,2,3+], Eoin Griffin[1,2+], Victor Guarochico-Moreira[1,2,4], Donnchadh Barry[1], Benhao Xin[1,2], Shiqi Huang[1,2], Andre K. Geim[1,2], Francois. M. Peeters[5], Marcelo Lozada-Hidalgo[1,2*]

[1]National Graphene Institute, The University of Manchester, Manchester M13 9PL, UK

[2]Department of Physics and Astronomy, The University of Manchester, Manchester M13 9PL, UK

[3]College of Advanced Interdisciplinary Studies, National University of Defense Technology, Changsha, Hunan 410073, China

[4]Escuela Superior Politécnica del Litoral, ESPOL, Facultad de Ciencias Naturales y Matemáticas, P.O. Box 09-01-5863, Guayaquil, Ecuador

[5]Departement Fysica, Universiteit Antwerpen, Groenenborgerlaan 171, B-2020 Antwerp, Belgium

*marcelo.lozadahidalgo@manchester.ac.uk

+These authors contributed equally

Experimental Section

Device fabrication. Apertures 10 µm in diameter were etched into silicon nitride substrates (500 nm SiN_x on B-doped Si, purchased from Inseto Ltd.), following the protocol previously reported[1]. Au electrodes were fabricated onto the substrates using photo-lithography and electron-beam evaporation. Mechanically exfoliated (monocrystalline) graphene[2] was then suspended over the apertures and on the Au electrodes (Figure S1). Pt nanoparticles were deposited by electron beam evaporation on the graphene film[3]. The discontinuous Pt film had a nominal thickness of 1 nm and is not electron conductive, which allowed using graphene as the electrode. Ranging the film thickness between 0.3 nm and 2 nm had no noticeable impact on the device performance. Thicker Pt films became electrically conductive, which shorted the graphene electrode. Thinner films displayed low proton currents. On the opposite side of the graphene film, an anion-exchange ionomer solution (Fumion FAA-3-SOLUT-10, FuMA-Tech purchased from Ion Power GmbH) was drop cast. The polymer acts as a versatile anion conductor in which both the type of charge carriers and their concentration can be controlled by placing it in contact with an electrolyte solution[4]. The polymer was placed in contact with an Ar-saturated alkaline electrolyte consisting of 1M KCl with KOH to set the pH of the electrolyte (typically 11). All measurements were carried out in a chamber with Ar environment and the electrolyte was saturated with Ar. Before measurements, the polymer side of the devices was left in contact with deionised water for several hours to remove impurities and was then exposed to the electrolyte solution.

The polymer and the Pt nanoparticles are not expected to display a photo-response, however, we corroborated this directly (Fig. S6). To that end, silicon nitride substrates with 10 µm diameter through holes were fabricated as discussed above. Fumion polymer was drop cast on one side. The opposite side was then decorated with Pt nanoparticles deposited by electron beam evaporation and then coated with Fumion (Fig. S6a). The devices were measured in dark conditions and solar simulated illumination. No photo-response was observed, as expected (Fig. S6b).

Electrical measurements. For electrical measurements the graphene electrodes were connected in a three-electrode geometry using a Pt counter electrode and an Ag/AgCl reference electrode. A dual channel Keithley SourceMeter 2636A that was programmed to function as a potentiostat was used to measure the potential of the graphene electrode vs the Ag/AgCl (voltmeter channel) and the current between graphene and the Pt electrode (source channel). During the measurement, a feedback unit (a Proportion Integration control loop) between the voltmeter and source channels set the potential vs Ag/AgCl reference into a required setpoint. For reference, we also performed measurements using an Ivium CompactStat.h potentiostat, which gave the same results. We typically scanned $\pm$150 mV around the zero current potential (vs the reference electrode) at sweep rates of 0.01 V min^{-1}. The photo-response of the devices in this work was characterised using a calibrated Newport Oriel Sol3A light source that produced solar-simulated illumination with maximum intensity of 100 mW cm^{-2}. The illumination intensity was controlled with the solar simulator's aperture diaphragm.
An important consequence of the small size of the graphene electrode is that the bulk electrolyte resistance does not limit the current measured[5,6]. For a cell with a microelectrode of radius r and electrolyte conductivity κ, the limiting resistance (R) is given by $R = (4\pi\kappa r)^{-1}$. In our measurements, $\kappa \approx$ 0.1 S cm^{-1}, $r = 5$ µm and hence $R \approx 1.6$ kΩ, which is at least 2 orders of magnitude smaller than any of those reported with graphene electrodes.

Faradaic efficiency measurements. Figure S4 shows a schematic of the experimental setup for Faradaic efficiency measurements, demonstrated in refs.[3,7]. The graphene device separated two chambers. The Pt-decorated side facing the inside of a chamber evacuated and connected to the mass spectrometer. The opposite side of the device faced the electrolyte solution. The hydrogen flux and electric current were measured simultaneously using a mass spectrometer (Inficon UL200 Detector) and a Keithley 2636A sourcemeter. We did this measurement in two ways. First, we applied a fixed voltage and illuminated the device in on-off cycles (Figure S4b). Second, the illumination was turned on and the voltage was swept (Figure S4c). Both methods yielded the same dependence of hydrogen flux versus current density, $\Phi_{H2} = I/2F$, with F the Faraday constant.
Figure S5a shows a schematic of our oxygen flux setup, which was demonstrated in ref.[7]. In brief, a graphene device was clamped to a transparent acrylic container with the polymer side of the device facing the inside of the container. The container had three gasket-sealed outlets for a Clark oxygen microelectrode (UNISENSE, OX-NP), a needle connected with an Argon supply and a Pt wire electrode. A small magnetic stir bar kept at a rotation rate of 300 rpm promoted gas convection in the electrolyte solution. The solution was purged with argon gas through the needle for at least 30 mins and the whole container was placed inside a chamber with constant argon gas circulation to prevent oxygen leakage into the cell. Electrical current and oxygen concentration [O_2] in the solution were measured simultaneously. In a typical measurement, voltage is applied to the device, illumination is turned on and then both illumination and voltage are turned off. Figure S5c shows d[O_2]/dt from a typical measurement. The area-normalised oxygen concentration $\Phi_O = (d[O_2]/dt)/A$ was correlated with the measured current via the Faradaic relation as $\Phi_O = I/4F$.
The functioning of the oxygen sensor was described in detail in ref.[7]. In brief, the sensor consists of a pipette containing an oxygen-reducing cathode, a reference electrode and a guard electrode. The tip of the sensor is sealed with a silicone membrane, which is impermeable to all ion but highly permeate to

gases[8]. This creates a chamber with a stable environment for the electrolyte (Figure S5b). During operation, the potential of the sensing cathode is polarized against the reference electrode and the diffusion of oxygen through the membrane is detected by the sensing cathode, via the oxygen-reducing reaction: $O_2 + 2H_2O + 4e^- \rightarrow 4OH^-$. The resulting pA-level current signal is amplified to convert it to voltage in the mV range. Since the sensing cathode only consumes a negligible amount of oxygen, the guard cathode removes the excess oxygen in the electrolyte.

Supplementary discussion

Wien effect. In a recent work, we reported that the proton currents arising from interfacial water dissociation through graphene electrodes are exponentially accelerated with increasing *E*[7]. However, we note that graphene's proton conductivity and the Wien effect are independent phenomena. Strong electric fields accelerate the water dissociation reaction, yielding additional protons and hence higher proton currents – a process modelled by Onsager's theory of the Wien effect. In ref. 7, we showed that this generation of additional protons fully explains the field dependence of the water dissociation in graphene electrodes and, hence, that the field effect is not due to an increase in graphene's proton conductivity in strong *E*. Moreover, we note that the proton conductivity of graphene is not expected to depend on the electric field[1]. This is because proton transport is determined by the total density of electron clouds in graphene[1]; whereas, even at the highest doping density used in our experiments, the number of electrons induced by *E* present only a small portion (<1%) of the total number of electrons in un-doped graphene[7].

Timescales. Our experiments provide insights into the timescales of the water dissociation process through graphene as follows. In our devices, illumination accelerates proton transfer via the photo-proton effect[3]. This process relies on the ability of graphene to absorb photons to excite electrons above the Fermi energy – the so-called hot electrons. The timescale during which the electrons remain excited, or hot, is in the pico-second timescale[3]. On the other hand, proton currents in our devices arise from water dissociation, which involves the separation of protons from hydroxide ions across graphene. Hence, the observation of photo-accelerated water dissociation in our devices suggests that the time it takes to separate the proton-hydroxide ion pairs through graphene is comparable to or faster than the lifetime of the hot electrons – otherwise the two processes would be decoupled and no photo-effect would be measured. On this basis, we suggest that the proton-hydroxide separation process takes place in the pico- or sub-picosecond timescale. For reference, we note that, as an independent approximation, the proton-hydroxide ion separation process can be expected to take place within timescales comparable to those of proton transport and proton-OH^- recombination in water (sub-picosecond timescale, ref.[9]). This independent approximation is consistent with the timescale estimated from the observation of the photo-effect.

The fast water dissociation ($H_2O \leftrightarrows H^+ + OH^-$) process eventually leads to full electrolysis ($H_2O \rightarrow H_2 + ½O_2$), producing hydrogen and oxygen gas. The gas evolution rates are slower than the dissociation rate and are generally expected to take much longer than the ps lifetime of hot electrons in graphene[3]. If these

reactions were limiting in our devices, we would not observe a photo-response. However, in our devices these reactions take place in the large Pt nanoparticle film (H_2 evolution) and the Pt counter-electrode (O_2 evolution), which are several orders of magnitude larger than the graphene electrode. In previous work we showed that because of this large size difference, the H_2 evolution rate is no longer limiting and the Pt film effectively behaves as drain reservoirs for protons[3]. The observation of a strong photo-response in the water electrolysis reaction in the present work shows that the same holds for the large Pt counter-electrode for O_2, as expected.

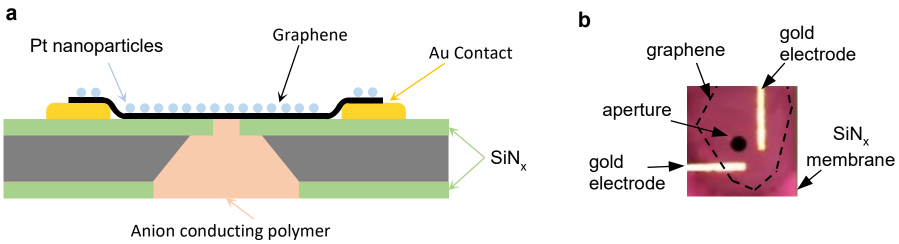

Figure S1. Device geometry. (a) Schematic of graphene electrode devices. (b) Optical image of one of these devices (plan view). Black circle, 10 µm diameter aperture in the SiN_x substrate. Dashed lines mark the area covered by monolayer graphene. Devices typically had two gold electrodes (usually they were shorted) in order to ensure good electrical contact with graphene.

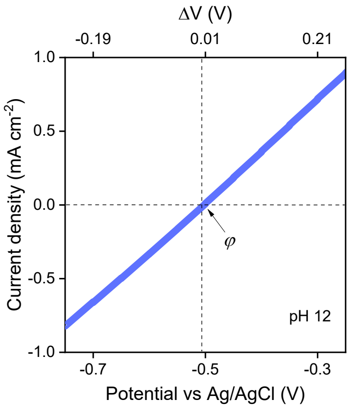

Figure S2. Current voltage characteristics. *I*-*V* response of devices vs reference electrodes. The potential at zero current, φ, is typically negative, in agreement with previous work[7]. The I-V response is linear for small $\Delta V = V-\varphi$ (top x-axis). Dashed lines, guide to the eye.

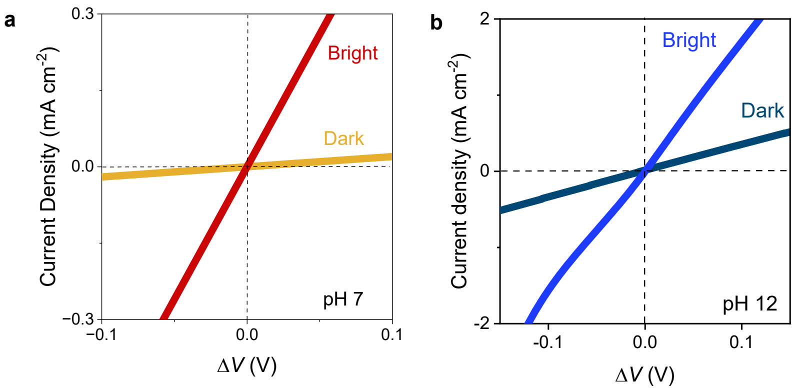

Figure S3. Photo-effect measured using different electrolyte pH. Examples of *I-V* characteristics of graphene electrode devices measured in dark and bright conditions using electrolyte pH 7, (a) and pH 12, (b) Solar simulated illumination of 100 mW cm^{-2}. The absolute value of the current depends strongly on pH, as reported in ref.[7], but the bright current increases by an order of magnitude with respect to the dark in all cases.

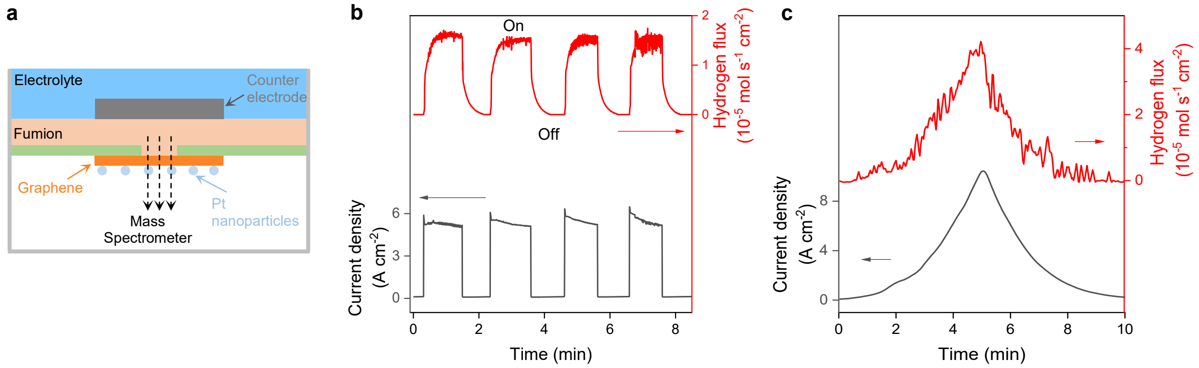

Figure S4. Hydrogen mass transport experiment. (a) Schematic of the experimental setup. (b) Example of current density and hydrogen flux measurements recorded simultaneously while switching the illumination on and off. *V*-bias, 1.8 V. (c), Example of current density and hydrogen flux measurements recorded simultaneously with illumination turned on, while sweeping bias voltage (0-2 V). pH 11.

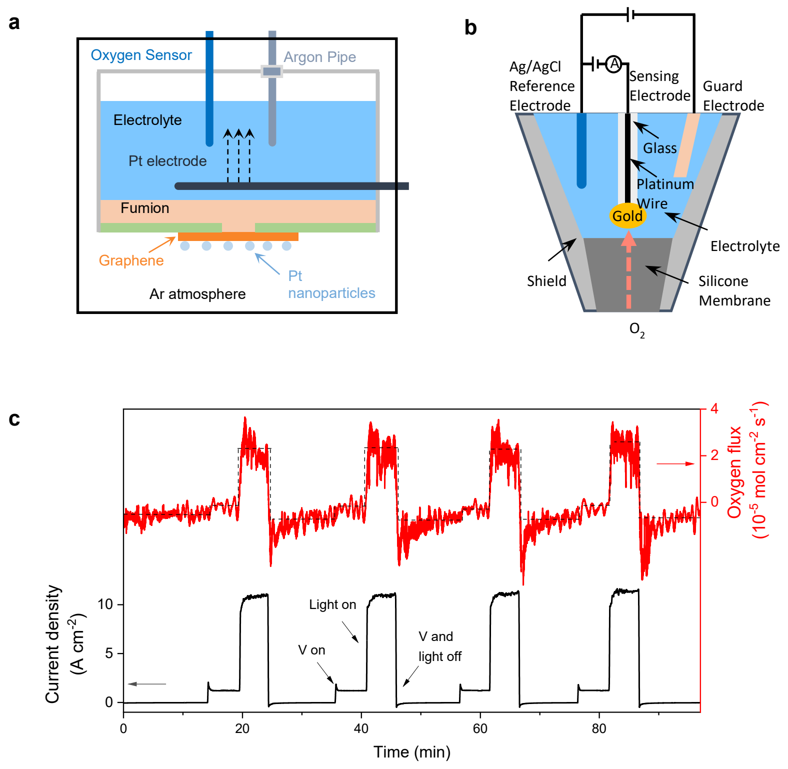

Figure S5. Oxygen mass transport experiment. (a) Schematic of the experimental setup. (b) Schematic of the oxygen sensor. (c) Example of current density and oxygen flux data recorded simultaneously while switching illumination on and off, pH 11. *V*-bias, 2.1 V. Measuring sequence includes four steps: zero bias in dark; voltage applied in dark; illumination switched on; both voltage and illumination switched off. Dotted lines, guide to the eye.

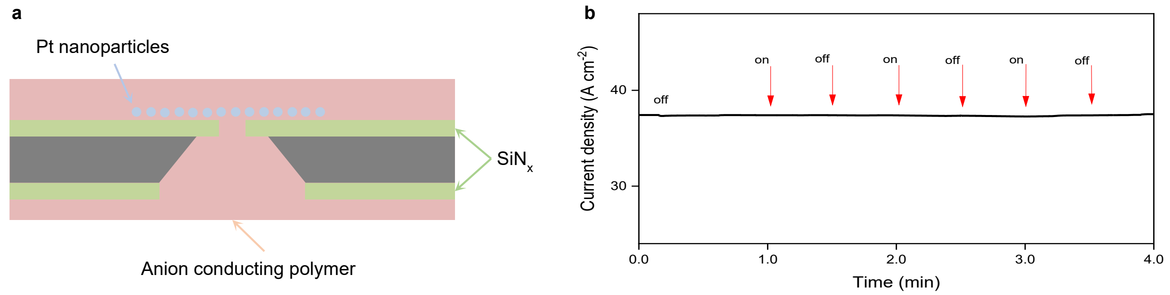

Figure S6. Absence of photo-response in devices without graphene. (a) Devices without graphene used to test the photo-response of the polymer and Pt nanoparticles. (b), Current density vs time for a device without graphene electrode in dark conditions and under solar-simulated illumination. The light was turned on and off in 30-second intervals (marked with red arrows). Voltage bias, 0.2 V. No photo-response was observed.

References

(1) Hu, S.; Lozada-Hidalgo, M.; Wang, F. C.; Mishchenko, A.; Schedin, F.; Nair, R. R.; Hill, E. W.; Boukhvalov, D. W.; Katsnelson, M. I.; Dryfe, R. A. W.; Grigorieva, I. V.; Wu, H. A.; Geim, A. K. Proton Transport through One-Atom-Thick Crystals. *Nature* **2014**, *516* (7530), 227–230. https://doi.org/10.1038/nature14015.

(2) Kretinin, A. V.; Cao, Y.; Tu, J. S.; Yu, G. L.; Jalil, R.; Novoselov, K. S.; Haigh, S. J.; Gholinia, A.; Mishchenko, A.; Lozada, M.; Georgiou, T.; Woods, C. R.; Withers, F.; Blake, P.; Eda, G.; Wirsig, A.; Hucho, C.; Watanabe, K.; Taniguchi, T.; Geim, A. K.; Gorbachev, R. V. Electronic Properties of Graphene Encapsulated with Different Two-Dimensional Atomic Crystals. *Nano Lett.* **2014**, *14* (6), 3270–3276. https://doi.org/10.1021/nl5006542.

(3) Lozada-Hidalgo, M.; Zhang, S.; Hu, S.; Kravets, V. G.; Rodriguez, F. J.; Berdyugin, A.; Grigorenko, A.; Geim, A. K. Giant Photoeffect in Proton Transport through Graphene Membranes. *Nat. Nanotech.* **2018**, *13* (4), 300–303. https://doi.org/10.1038/s41565-017-0051-5.

(4) Merle, G.; Wessling, M.; Nijmeijer, K. Anion exchange membranes for alkaline fuel cells: A review. *J. Memb. Sci* **2011**, *377* (1–2), 1–35. https://doi.org/10.1016/j.memsci.2011.04.043.

(5) Bard, A. J.; Faulkner, L. R. *Electrochemical Methods: Fundamentals and Applications*, 2nd ed.; Wiley: New York, 2001; pp 104-106

(6) Heinze, J. Ultramicroelectrodes in Electrochemistry. *Angew. Chem. Int. Ed. Engl.* **1993**, *32* (9), 1268–1288. https://doi.org/10.1002/anie.199312681.

(7) Cai, J.; Griffin, E.; Guarochico-Moreira, V.; Barry, D.; Xin, B.; Yagmurcukardes, M.; Zhang, S.; Geim, A. K.; Peeters, F. M.; Lozada-Hidalgo, M. Wien Effect in Interfacial Water Dissociation through Proton-Permeable Graphene Electrodes. Nat. Commun. **2022 (13)**, 5776. https://doi.org/10/1038/s41467-022-33451-1

(8) Hwang, S.-T.; Tang, T. E. S.; Kammermeyer, K. Transport of Dissolved Oxygen through Silicone Rubber Membrane. *J. Macromol. Sci. B* **1971**, *5* (1), 1–10. https://doi.org/10.1080/00222347108212517.

(9) Hassanali, A.; Prakash, M. K.; Eshet, H.; Parrinello, M. On the Recombination of Hydronium and Hydroxide Ions in Water. *Proc. Natl. Acad. Sci. U. S. A* **2011**, *108* (51), 20410–20415. https://doi.org/10.1073/pnas.1112486108.
